# Reassessing the Reassessment of suPAR in Glomerular Disease

**DOI:** 10.3389/fmed.2014.00059

**Published:** 2015-01-14

**Authors:** Björn K. I. Meijers, Jochen Reiser

**Affiliations:** ^1^Department of Nephrology, UZ Leuven, Leuven, Belgium; ^2^Department of Microbiology and Immunology, KU Leuven, Leuven, Belgium; ^3^Department of Medicine, Rush University Medical Center, Chicago, IL, USA

**Keywords:** suPAR, FSGS, glomerular disease, mouse models, GFR

Soluble urokinase receptor (suPAR) is proposed as circulating factor in focal and segmental glomerulosclerosis (FSGS) (1). Spinale et al. attempt to validate the role of suPAR in glomerular disease (2). Their mouse overexpression experiments of physiological suPAR forms are distinct from the studies by Wei et al. that expressed either alternate suPAR forms or used different mouse models (1). Additional experiments will help to further clarify the distinct roles of suPAR variants.

Spinale et al. extend previous clinical studies indicating that glomerular filtration rate (GFR) is an important determinant of suPAR (3). This, however, does not imply that suPAR is only bystander, as illustrated by epidemiological studies linking elevated suPAR – independent of the eGFR – to cardiovascular disease in patients with CKD (4).

As for most large protein plasma components, the balance between generation and clearance determines suPAR accumulation, adding complexity when studying the direct renal effects of suPAR, especially when possibly also causing kidney disease. Biopsy proven FSGS patients with GFR >40 ml/min have in 50% elevated suPAR levels (5), suggesting also suPAR production. In addition, suPAR fragments (measured and unmeasured) may cause podocyte injury, potentially contributing to a reduced GFR. The strong effect of a low GFR on serum suPAR levels in observational studies could obfuscate the effects of suPAR-induced glomerular pathology.

In conclusion, dependence of serum suPAR levels on GFR precludes using suPAR as a single value biomarker for FSGS in conditions of low GFR, but this correlation does not serve as an explanation for elevated suPAR under preserved GFR (Figure 1).

**Figure 1:**
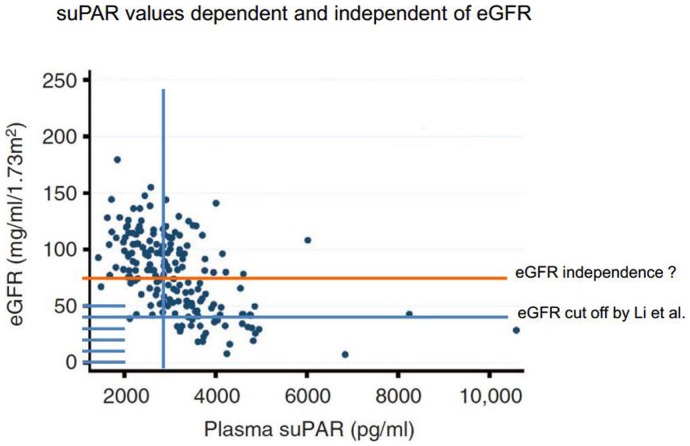
**suPAR plasma levels above cut off of 3000 pg/ml in patients with eGFR >40 ml/min (blue line) as suggested by Li et al. applied to the patient cohort by Spinale et al**. These values are likely not explained by reduced GFR alone. Elevated suPAR level in preserved renal function patients (orange line) is likely not dependent on eGFR.

## Conflict of Interest Statement

Jochen Reiser is an inventor on pending and issued patents related to anti-proteinuric therapies. He stands to gain royalties from their present and future commercialization. He is also a co-founder and advisor to TRISAQ, a biotechnology company. The Associate Editor Nada Alachkar declares that, despite having previously published with Jochen Reiser, the review process was handled objectively and no conflict of interest exists. Björn K. I. Meijers declares no conflict of interest.
